# Functional characterization of a novel non**-**coding mutation “Ghent +49A > G” in the iron-responsive element of L-ferritin causing hereditary hyperferritinaemia-cataract syndrome

**DOI:** 10.1038/s41598-017-18326-6

**Published:** 2017-12-21

**Authors:** Stijn Van de Sompele, Lucie Pécheux, Jorge Couso, Audrey Meunier, Mayka Sanchez, Elfride De Baere

**Affiliations:** 1 Department of Medical Genetics, Ghent University Hospital, Ghent, Belgium; 2 Department of Pediatrics, Centre Hospitalier Universitaire Saint-Pierre, Brussels, Belgium; 3 Program of Predictive and Personalized Medicine of Cancer, Germans Trias and Pujol Research Institute (PMPPC-IGTP), Campus Can Ruti, Badalona, Spain; 4 Iron metabolism: regulation and diseases group, Josep Carreras Leukaemia Research Institute (IJC), Campus ICO - Germans Trias i Pujol, Badalona, Spain; 5 Department of Ophthalmology, Centre Hospitalier Universitaire Saint-Pierre, Brussels, Belgium

## Abstract

Hereditary hyperferritinaemia-cataract syndrome (HHCS) is a rare disorder usually caused by heterozygous mutations in the iron-responsive element (IRE) in the 5′ untranslated region (5′UTR) of the L-ferritin gene (*FTL*), disturbing the binding of iron regulatory proteins (IRPs) and the post-transcriptional regulation of ferritin expression. Here, the proband of a consanguineous family displayed moderate bilateral cataracts and elevated serum ferritin in the absence of iron overload. The parents and siblings showed variable degrees of mild bilateral cataracts combined with elevated levels of circulating ferritin. Sequencing of *FTL* identified a novel 5′UTR mutation c.-151A > G, also named “Ghent +49A > G”. The zygosity of the mutation, occurring in homozygous and heterozygous state in the proband and other affected family members respectively, correlated well with severity of ophthalmological and hematological manifestations. The substitution is expected to impair the secondary structure of the upper IRE stem. Functional characterization of +49A > G by electrophoretic mobility shift assays demonstrated a reduced binding affinity for IRP1 compared to the wild-type IRE of *FTL*. Overall, we have expanded the repertoire of deleterious biallelic *FTL* IRE mutations in HHCS with this novel +49A > G mutation, the zygosity of which correlated well with the disease expression.

## Introduction

Hereditary hyperferritinaemia-cataract syndrome (HHCS) (OMIM 600886) is a rare autosomal dominant disease (estimated prevalence of 1 in 200,000), characterized by the combination of elevated serum ferritin in the absence of iron overload or other hematological abnormalities and progressive cataracts of a highly distinctive morphology^1^. The genetic disorder was first described in 1995 by two independent groups^2,3^. HHCS might be misdiagnosed as hereditary hemochromatosis, since serum ferritin levels are still widely used to identify hereditary and acquired iron overload conditions^4^. As a consequence, patients with HHCS could undergo inadequate follow-up with repeated exams in search of iron overload or even an inappropriate phlebotomy treatment that leads to iron deficiency anemia, stressing the importance of a correct clinical diagnosis of HHCS^3^.

Patients with HHCS present a disease-causing mutation in the *FTL* gene, located on chromosome 19 (19q13.1). The *FTL* gene encodes the light subunit, L-ferritin (19 kDa), that together with the heavy subunit, H-ferritin (21 kDa, encoded by the *FTH* gene located on chromosome 11q12.3) constitutes the 24-subunit heteropolymer ferritin protein^5^. Ferritin is an ubiquitously expressed protein responsible for the storage and intracellular distribution of iron. The expression of the ferritin subunits is modulated by intracellular iron levels through a tightly regulated negative feedback system based on the interaction between cytoplasmic iron-sensing mRNA-binding proteins, the iron regulatory proteins (IRP1 and IRP2), and a non-coding *cis-*acting stem-loop structure located in the 5′ untranslated region (5′UTR) of both L- and H-ferritin mRNA, the iron-responsive elements (IREs)^6^. These IREs comprise four important structural parts: the hexanucleotide apical loop, the upper and lower stem consisting of palindromic sequences and the cytosine bulge, an unpaired cytosine residue (C-bulge) located in between the upper and lower stem^7^. In conditions of low intracellular iron levels, the high affinity IRP/IRE binding prevents the recruitment of the small ribosomal subunit to the mRNA. As a consequence, the synthesis of the ferritin subunits is repressed at the translational level which limits the storage and utilization of cytoplasmic iron. Conversely, when intracellular iron levels increase the IRP/IRE binding affinity decreases due to conformational changes of IRP1 and the proteasomal degradation of IRP2, resulting in the initiation of ferritin mRNA translation and the consequent upregulation of ferritin synthesis^8,9^.

Heterozygous mutations in the IRE of *FTL* mRNA disturbing cellular iron homeostasis are characteristic of HHCS. *FTL* IRE mutations reduce the IRP/IRE binding affinity due to changes in the IRE secondary structure and/or the stability of the IRE^10^, abrogating the IRP/IRE interaction and leading to uncontrolled translation of *FTL* mRNA, independent of the iron status. The constitutive overproduction of L-ferritin gives rise to L-subunit rich ferritin heteropolymers and ferritin homopolymers of 24 L-subunits, resulting in both hyperferritinaemia and intracellular ferritin accumulation^11^. Although ferritin accumulates in all cell types of HHCS patients, it turns out to be toxic only in the crystallin-containing lens fiber cells. There, crystalline L-ferritin deposits can be found, which may affect the solubility of other lens proteins or induce oxidative lens damage, altogether leading to the formation of cataracts^12^.

Here, our aim was to molecularly explain the variable expressivity of hyperferritinaemia and bilateral cataracts in different members of a consanguineous family with HHCS, and to functionally characterize a novel mutation in the 5′UTR of *FTL*.

## Methods

### Clinical assessment of patients

**Figure 1:**
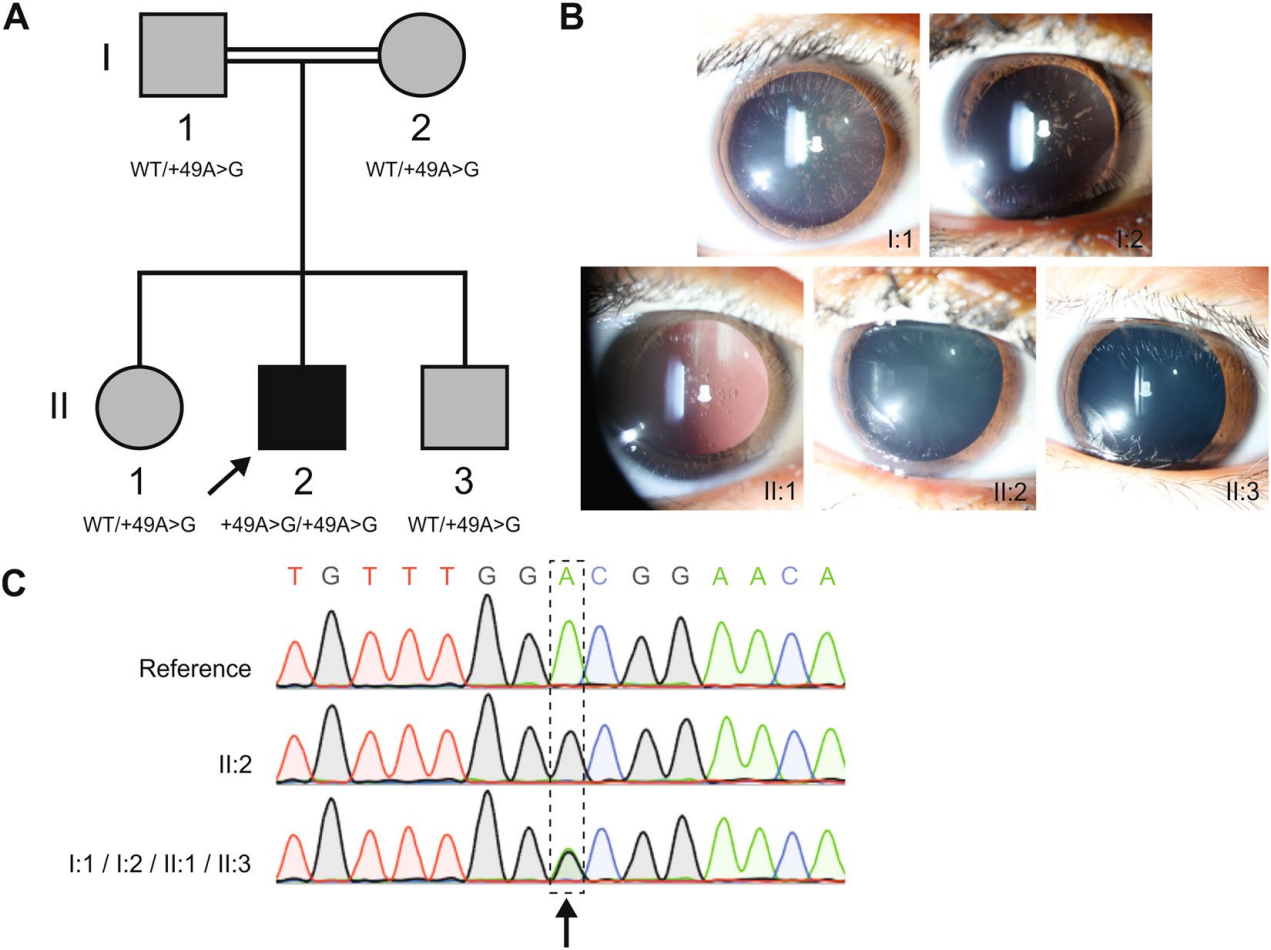
Overview of the HHCS-affected family. (**A**) Pedigree of the family investigated in this study, with segregation of the Ghent +49A > G mutation. The severity of the phenotype is represented by the darkness of the pedigree symbol. The pedigree shows parental consanguinity between I:1 and I:2. The proband II:2 is indicated by an arrow. (**B)** Slit-lamp examination of the anterior segment of the proband (II:2) displayed moderate central nuclear bilateral cataracts, in the absence of scattered flecks. The proband’s father (I:1) presented with mild bilateral central nuclear cataracts with typical distinctive peripheral spicules, while the mother (I:2) and siblings (II:1, II:3) demonstrated mild bilateral central nuclear cataracts with less dense opacities. (**C**) Electropherograms of Sanger sequencing of the IRE located in the 5′UTR of the *FTL* gene, using genomic DNA of patients. The arrow indicates the nucleotide change +49A > G (c.-151A > G, NM_000146.3). The WT sequence is displayed on top as a reference, the homozygous sequence (II:2) in the middle and the heterozygous sequence (I:1, I:2, II:1, II:3) at the bottom.

A Belgian family of Moroccan descent with parental consanguinity (second cousins) (Fig. 1A) underwent hematological and ophthalmological assessment following a clinical diagnosis of HHCS in the 8-year-old proband (II:2). In particular, slit-lamp biomicroscopy and the measurement of iron parameters were performed. Normal serum ferritin levels are ranging from 12 to 300 µg/L for males and from 12 to 150 µg/L for females^13^. An extended pedigree, illustrating the consanguineous relationship in this family, can be found in Supplementary Figure 1.

### *FTL* analysis

The proband (II:2), the parents (I:1, I:2) and two siblings (II:1, II:3) were referred to the Center for Medical Genetics in the Ghent University Hospital for genetic testing of the *FTL* 5′UTR. An informed consent was obtained from all subjects or their legal representative. Genomic DNA was extracted from peripheral blood using the QIAamp DNA blood mini kit, according to manufacturer’s instructions (Qiagen). In our standard workflow the coding regions, exon-intron boundaries and UTRs of the *FTL* gene (NM_000146.3) were PCR amplified starting with 40 ng of genomic DNA using four primer pairs (Supplementary Table 1). Purification of the PCR products by Exonuclease I and Antarctic Phosphatase (New England Biolabs) treatment was followed by bi-directional Sanger sequencing using the BigDye Terminator v3.1 Cycle Sequencing Kit (Applied Biosystems). Sequencing products were purified by CleanDTR magnetic beads (GC Biotech) and analyzed on a ABI PRISM 3730XL Sequencer (Applied Biosystems). Data-analysis and variant calling was performed with the SeqScape Software (Applied Biosystems). Given the clinical diagnosis of HHCS, data analysis was confined to the 5′UTR of *FTL*. Variants were designated according to the HGVS nomenclature and the classical nomenclature used for HHCS variants. Variant classification was performed following the American College of Medical Genetics (ACMG) guidelines of 2015^14^. The study was conducted in accordance with the tenets of Helsinki and was approved by the Ghent University Hospital Ethics Committee (approval 2004/094).

### Confirmation of homozygosity

To exclude false homozygosity of the 5′UTR mutation in the proband due to an *FTL* deletion in *trans* with the mutation, three qPCR primer pairs were used, located in the *FTL* 5′UTR region (Supplementary Table 1). Copy number analysis was performed on the LightCycler 480 real-time PCR instrument (Roche) using the SYBR Green Master Mix (Roche) and 20 ng of genomic DNA of each patient and three positive controls^15^. Each reaction was run in duplicate. Data analysis was executed using the qBase+ software (Biogazelle) and results were normalized to two reference assays (*ZNF80*, *GPR15*).

### Genome-wide SNP genotyping and homozygosity mapping

In order to assess the size of the autozygous region and haplotype in which the homozygous *FTL* 5′UTR mutation is located, genome-wide SNP genotyping was performed using the HumanCytoSNP-12 BeadChip platform (Illumina). Plink software, integrated in ViVar, identified homozygous regions >1 Mb which were ranked according to their length and the number of SNPs, as described^16^.

### *FTL* RNA fold predictions

RNA folding analysis to predict the IRE structure of wild-type (WT) and mutated *FTL* was carried out using Mfold (http://unafold.rna.albany.edu/)^17^ and Sfold web server (http://sfold.wadsworth.org/)^18^. RNA sequences used for fold predictions are shown in Supplementary Table 2.

### Plasmid construction

Plasmids for generating RNA probes for electrophoretic mobility shift assay (EMSA) experiments were constructed based on the I-12.CAT plasmid^19^ using annealed synthetic complementary oligonucleotides corresponding to the sequence of interest. Constructs were made for the *FLT* 5′ IRE +49A > G mutation as well as the WT sequence (*FTL* 5′ IRE WT, negative control) and a mutant version bearing a C deletion in the apical loop (*FTL* 5′ IRE +39ΔC, positive control), not interacting with IRP1^20^. After ligation using the Quick Ligation kit (New England Biolabs), 2 μL of the ligation product was heat-shock transformed into 50 µL competent TOP10 bacterial cells (Invitrogen) which were plated on LB plates containing 100 μg/mL ampicillin. Colonies were picked and grown in LB broth. Plasmid DNA was isolated using the Nucleospin plasmid mini DNA purification kit (Macherey-Nagel). Sanger sequencing was performed (GATC Biotech, Konstanz) to verify the presence of the correct insert in each construct. All oligonucleotides used for cloning purposes are listed in Supplementary Table 3. Plasmids were linearized and used as template for T7 *in vitro* transcription (MEGAscript T7 kit, Life Technologies).

### EMSA experiments

Two types of EMSA experiments were performed in this study: direct and competitive EMSA. For direct EMSA experiments, fluorescent-labeled probes corresponding to *FTL* 5′ IRE WT, +39ΔC and +49A > G were incubated with the recombinant IRP1 protein^19^ in order to qualitatively assess IRP1/IRE binding. For competitive EMSA experiments, the fluorescent-labeled WT probe was incubated with recombinant IRP1 together with increasing amounts of non-labeled competitor *FTL* 5′ IRE WT, +39ΔC and +49A > G probes. The extent to which the non-labeled probes competed with the labeled WT probe for binding the IRP1 protein, provided a quantitative insight into IRP1/IRE binding affinity.

The T7 *in vitro* transcription reaction for the labeled probes was performed in 1X transcription buffer, 0.33 mM rATP, rCTP, rGTP, 3.33 μM rUTP, 20 μM ATTO-680 aminoallyl-rUTP (Jena Bioscience), 240 units T7 RNA polymerase (EMBL Heidelberg core facility), 60 units RNasin Ribonuclease Inhibitor (Promega), and 30 mM DTT in a total reaction volume of 45 µL, using 6 µg of template DNA. After incubation for 4 h at 37 °C, template DNA was degraded by TURBO DNase (Ambion) treatment. The non-labeled competitors were *in vitro* transcribed using the MEGAscript T7 kit (Life Technologies) according to the manufacturer’s instructions. ATTO-680 labeled probes and unlabeled competitors were purified by phenol/chloroform/isoamyl alcohol extraction and used as probes. Prior to protein binding, 100 ng of labeled probe, and increasing molar excess (1X, 2X, 5X, 10X, 20X, 40X, 80X) of unlabeled competitor probes in case of competitive EMSA, were denatured at 95 °C for 5 min, followed by renaturation at room temperature. Probes were then incubated with 200 ng recombinant IRP1 protein^19^ in CLB buffer (25 mM Tris/HCl pH 7.4, 40 mM KCl, 1% Triton X-100, 1X cOmplete EDTA-free protease inhibitor cocktail) for 15 min at room temperature. In order to displace non-specific RNA-protein interactions, 50 ng of sodium heparin was added to the reaction mix. After incubation for 10 min at room temperature, 2 μL of 80% glycerol loading buffer was added and reaction mixtures were loaded on a 5% native acrylamide (60:1 acrylamide:bisacrylamide) gel in 1X TBE buffer. Gels were run at 80 V at 4 °C and visualized in the 700 nm channel using the Odyssey Infrared Imaging System (LI-COR Biosciences), at an intensity setting of 7.0, with resolution set to 169 µm and focus offset to 1.0 mm. Adjustment of brightness and contrast levels was applied to the entire images using the ImageJ software. After applying the despeckle and background subtraction tool, absolute band intensities corresponding to IRP1/IRE complexes were calculated with the ImageJ software. For direct EMSA experiments, intensities were normalized relative to the intensity of the shifted band of WT IRE. Normalization of intensities of competitive EMSA experiments was done relative to the intensity of the band in lane N. Direct and competitive EMSA experiments were performed in quadruplicate and triplicate respectively.

### Statistical analysis

The results from the direct EMSA experiments were compared using one-way analysis of variance (ANOVA) with a *post hoc* Bonferroni multiple-comparison test. Differences between groups were considered statistically significant for p values less than 0.05. Statistical analyses were performed with GraphPad Prism 5.0.

### Calculation of apparent dissociation constants (K_Dapp_)

The calculation of the K_Dapp_ values was performed using a previously reported method for competition assays^21^. The plot of bound labeled WT probe against the concentration of free unlabeled competitor was best fit to the single independent site model:

1 $$f=1-\frac{{[C]}_{free}}{{[C]}_{free}+{K}_{Dapp}}$$

with *f* the ratio of observed intensity relative to the intensity in the absence of competitor, K_Dapp_ the apparent dissociation constant for the competitor under investigation, and [C]_free_ the concentration of the free competitor:

2 $${[C]}_{free}={[C]}_{i}-{[H]}_{i}(1-f)$$

with [C]_i_ the initial concentration of competitor, [H]_i_ the initial amount of labeled WT probe. The non-linear leastsquares best fit apparent dissociation constant was then calculated from Equation (1).

## Results

### Clinical findings

The 8-year-old proband (II:2) was referred because of highly elevated serum ferritin (2784 µg/L) without clinical, biological or radiologic evidence of iron overload. His familial history revealed consanguinity (Fig. 1A), a long follow-up for moderate hyperferritinaemia without iron overload in his mother (I:2) with negative genetic testing for *HFE*-associated hereditary hemochromatosis and early-onset of bilateral cataracts at age 30 in his father (I:1). Slit-lamp examination of the anterior segment of the proband (II:2) unveiled moderate bilateral cataracts (Fig. 1B) compatible with a clinical diagnosis of HHCS. In the proband’s parents (I:1, I:2) and siblings (II:1, II:3) variable degrees of mild bilateral cataracts (Fig. 1B) were observed, with elevated, but significantly lower levels of circulating ferritin compared to the proband (576 µg/L, 586 µg/L, 460 µg/L, and 661 µg/L respectively), with normal levels of serum iron and transferrin saturation. The father (I:1) was also diagnosed with possible Fuchs’ uveitis, a condition often resulting in secondary cataract^22^. In this case, however, the cataract morphology showed a different aspect than that usually observed in Fuchs’ uveitis. The biological and clinical findings in this family are summarized in Table 1.

**Table 1:** Hematology values of the proband (II:2), parents (I:1, I:2), and two siblings (II:1, II:3) and summary of their molecular findings.

	I:1	I:2	II:1	II:2	II:3	Reference values
Age/sex	38/M	35/F	13/F	8/M	6/M	—
Serum ferritin (µg/L)	576	586	460	2784	661	12–300 (M), 12–200 (F)
Serum iron (μg/dL)	117	83	155	101	65	50–170
Transferrin (mg/dL)	235	325	314	280	307	200–360
Transferrin saturation (%)	36	18	39	25	15	15–50
AST (mU/mL)	18	19	19	29	33	10–34
ALT (mU/mL)	17	12	12	16	15	10–40
Cataract	Mild cataracts	Mild cataracts	Mild cataracts	Moderate cataracts	Minimal cataracts	—
Other	Fuchs’ uveitis	—	—	—	—	—
Genotype	WT/+49A > G	WT/+49A > G	WT/+49A > G	+49A > G/+49A > G	WT/+49A > G	WT/WT

Values are compared with normal reference values. Abbreviations used: ALT: alanine aminotransferase; AST: aspartate aminotransferase; F: female; M: male; WT: wild type.

### Molecular genetic findings

Subsequent analysis of the 5′UTR of the *FTL* gene encoding the *FTL* IRE in the HHCS-affected proband revealed a novel homozygous mutation c.-151A > G (NM_000146.3). In agreement with the traditional nomenclature for *FTL* IRE mutations, the alternative name for this mutation is the “Ghent +49A > G” mutation (relative to the transcription initiation signal). Segregation analysis showed the presence of the Ghent +49A > G mutation in a heterozygous state in the parents as well as in both siblings (Fig. 1C). This variant was not reported in the consulted public databases (1000 Genomes Project, ExAC, dbSNP, HGMD). No other likely pathogenic variants in the *FTL* gene were observed in neither the proband nor the family members.

To exclude false homozygosity of the Ghent +49A > G mutation in the proband due to a deletion *in trans* with the mutation, a qPCR analysis was performed using three amplicons neighbouring the mutation. The corresponding results ruled out false homozygosity and confirmed that the Ghent +49A > G mutation in the proband is present on both alleles (Supplementary Figure 2). Following ACMG guidelines, this mutation was classified as a pathogenic mutation (class 5) (Supplementary Table 4).

Given the parental consanguinity, genome-wide SNP genotyping was performed in the proband, the parents and two siblings, in order to have more insight into the size of the autozygous region and corresponding haplotype containing the homozygous 5′UTR mutation. Homozygosity mapping in the proband revealed seven homozygous regions (>1 Mb) unique to the proband, one of which contains the *FTL* gene. An overview over the regions of homozygosity can be found in Supplementary Table 5.

### The Ghent +49A > G mutation is predicted to impair the IRE structure

**Figure 2:**
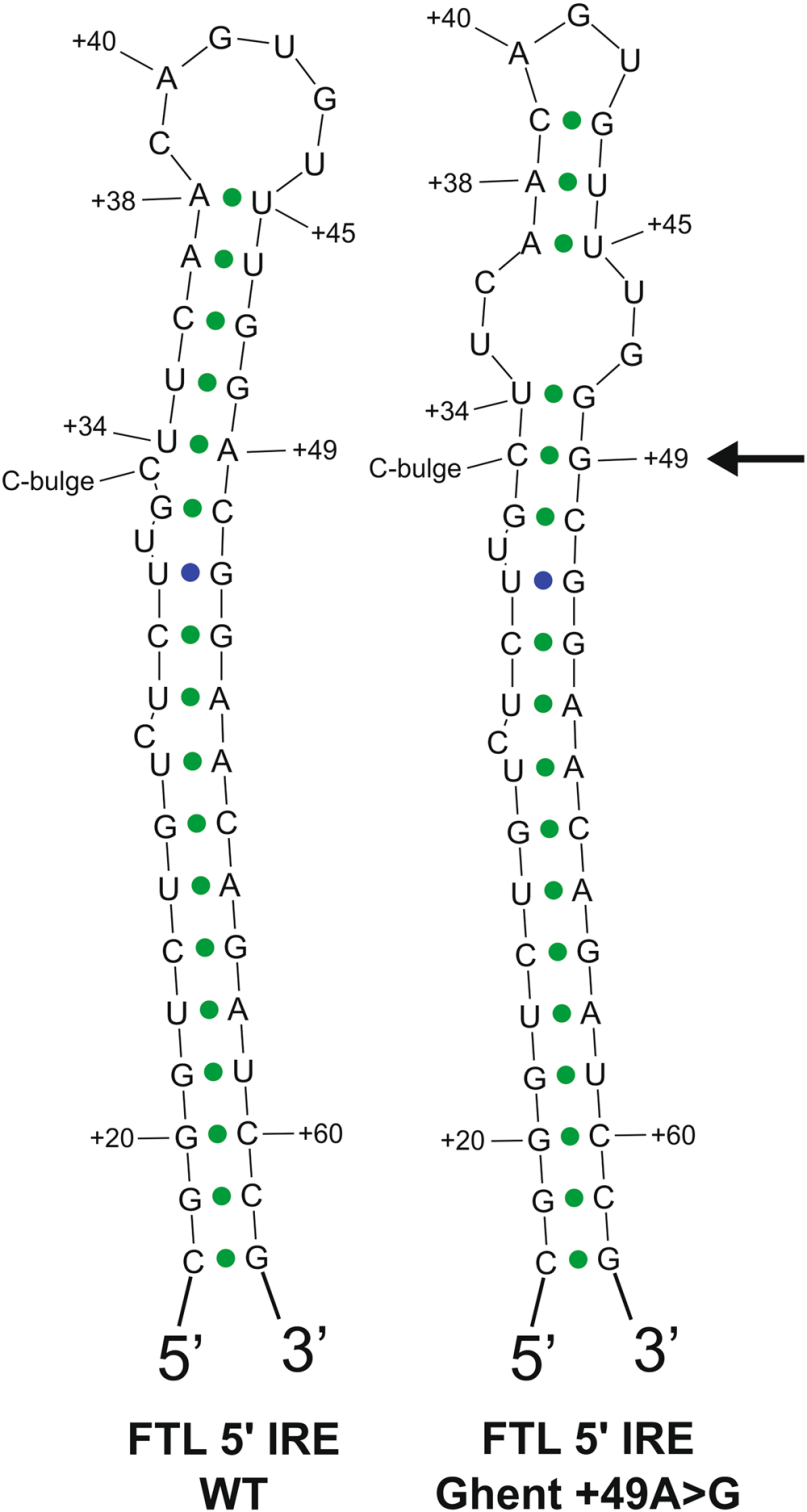
Predicted secondary structure of WT and +49A > G mutated *FTL* 5′UTR IRE RNA, computed using the Sfold web server^18^. The +49A > G mutation, indicated with an arrow, is expected to influence base pairing in the upper stem, resulting in an impaired secondary structure of the hexanucleotide loop and loss of the cytosine bulge. Secondary structure modeling by the Mfold web server yields the same result. Nucleotides are numbered from the transcription initiation signal. Green dots represent common base pairs between the ensemble centroid (EC) structure diagram and the minimum free energy (MFE) structure diagram. Blue dots represent base pairs predicted only by the MFE structure diagram.

RNA secondary structure modeling of WT and mutated *FTL* 5′ IRE sequences was performed using the Mfold and Sfold web server. Both folding software packages predict that the Ghent +49A > G mutation, which is located at the basis of the upper stem, just above the cytosine bulge, is likely to disturb the WT IRE conformation (Fig. 2). The substitution of adenine by guanine at position +49 is expected to affect base pairing with uracil at position +34, inducing a broader rearrangement of base pairing in the upper stem, an altered secondary structure of the apical loop and loss of the cytosine bulge.

### The Ghent +49A > G mutation shows reduced IRP1 binding

**Figure 3:**
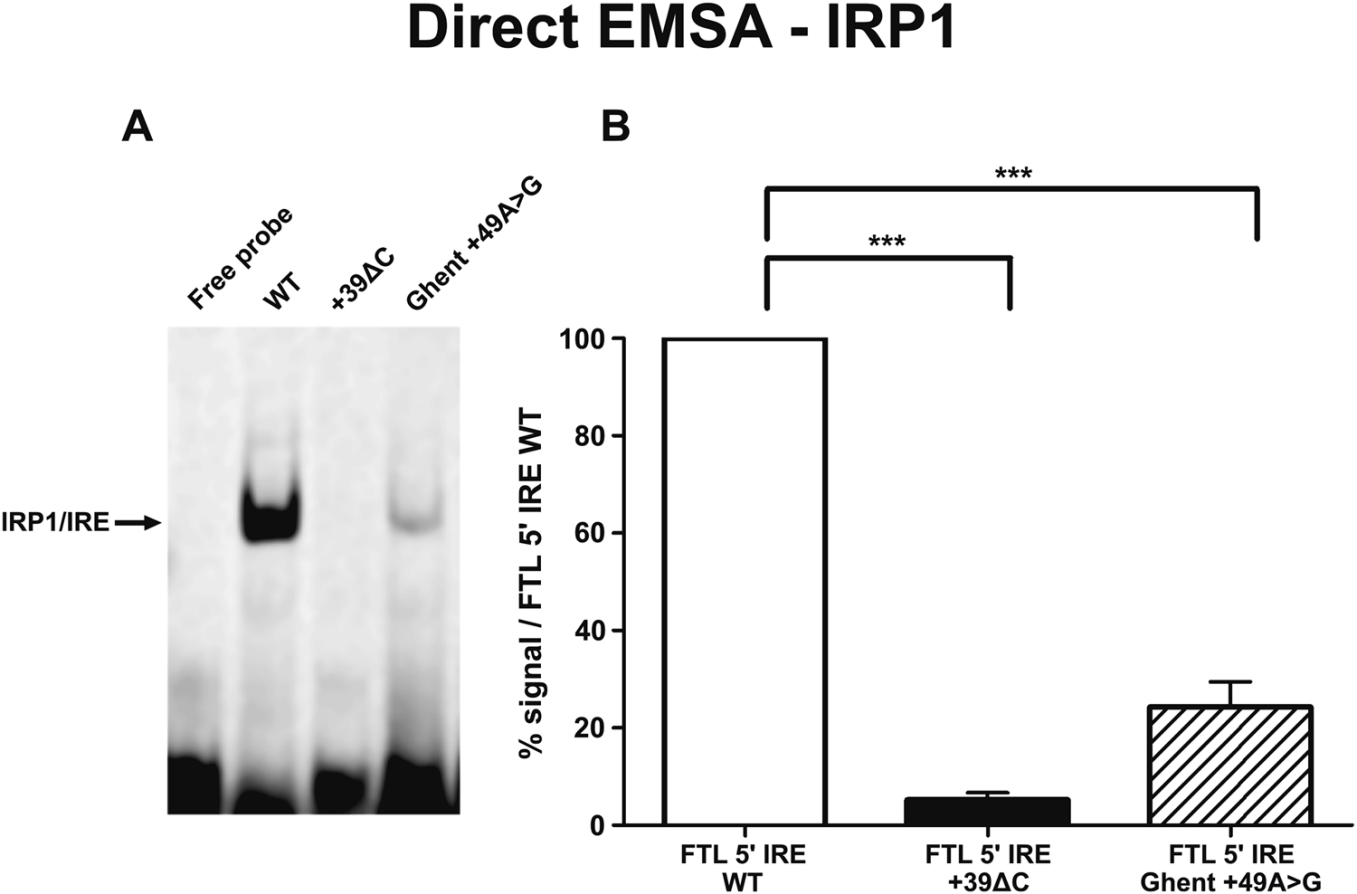
Direct EMSA experiments to qualitatively evaluate the effect of the Ghent +49A > G mutation on IRP/IRE binding affinity. Therefore, fluorescent-labeled probes of the +49A > G mutant IRE were incubated with IRP1 protein and loaded on a 5% native acrylamide gel. Fluorescent-labeled probes of the WT and +39ΔC mutant IRE were used as negative and positive controls respectively. (**A**) Representative gel image showing the intensities of the shifted IRP1/IRE complex. Lane 1 contains the labeled WT probes in absence of IRP1. Lane 2, 3 and 4 respectively contain labeled WT, +39ΔC mutant and +49A > G mutant probes in the presence of IRP1. (**B**) Band intensities of the two mutant groups were normalized relative to the intensity of the shifted band of WT IRE (lane 2) and presented as the mean of four experiments ± standard error of the mean. Statistical analysis was done by one-way ANOVA, followed by *post hoc* Bonferroni multiple comparison test (*p < 0.05, **p < 0.01, ***p < 0.001).

**Figure 4:**
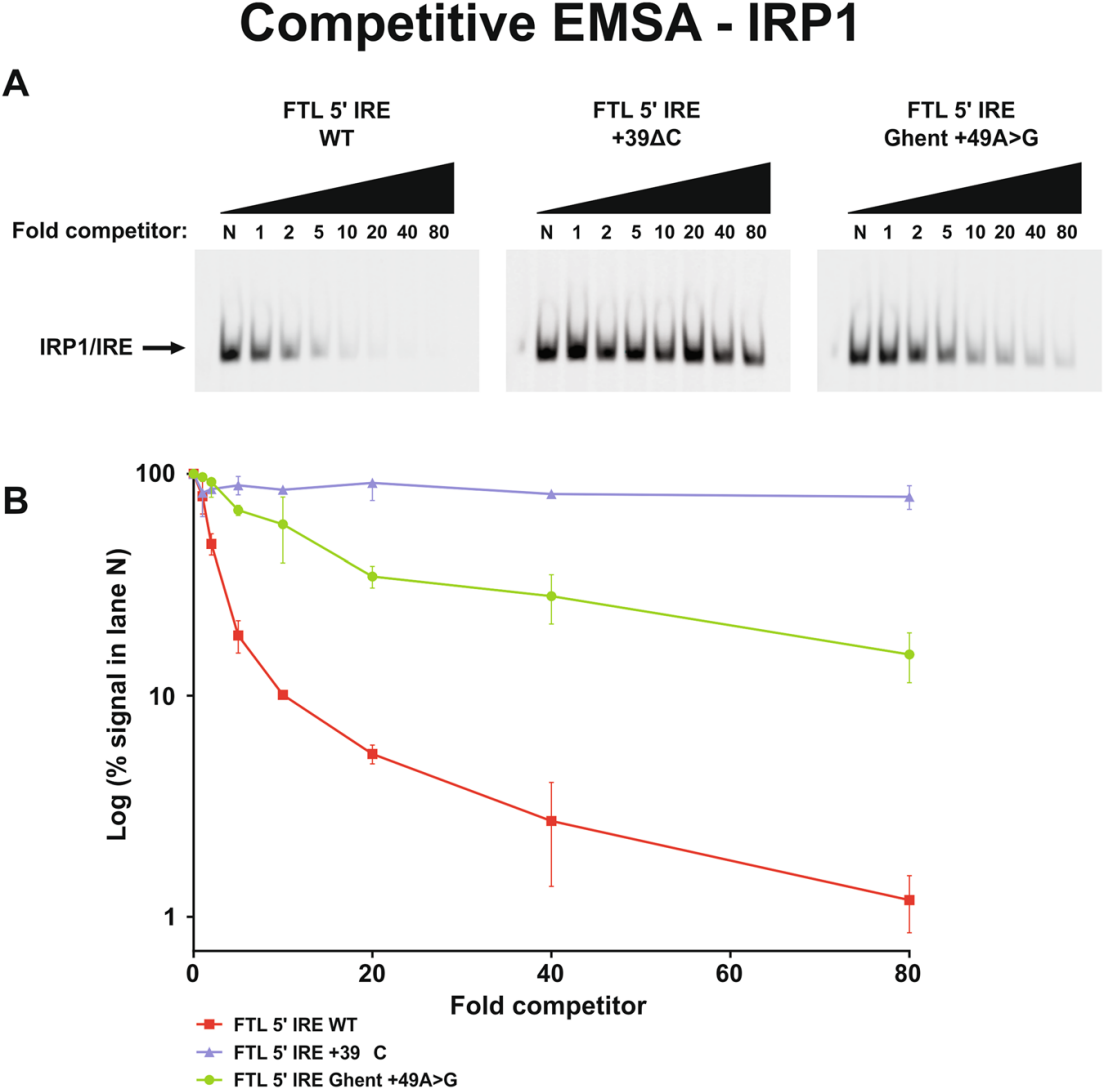
Competitive EMSA experiments to quantitatively evaluate the effect of the Ghent +49A > G mutation on IRP/IRE binding affinity. Fluorescent-labeled probes of the WT IRE were incubated with IRP1 protein and increasing molar excess (1X, 2X, 5X, 10X, 20X, 40X, 80X) of unlabeled +49A > G mutant competitor probes. Unlabeled competitor probes of the WT and +39ΔC mutant IRE were used as negative and positive controls respectively. Reactions were loaded on a 5% native acrylamide gel. (**A**) Representative gel images showing the intensities of the shifted IRP1/IRE complex. Lane N contains the labeled WT probe with IRP1 in the absence of unlabeled competitor. The following lanes contain labeled WT probe with IRP1 and increasing amounts of WT, +39ΔC mutant and +49A > G mutant competitor. (**B**) Band intensities of the WT and two mutant groups were normalized relative to the intensity of the shifted band in the absence of competitor (lane N) and presented on a logarithmic scale in function of the molar excess of the competitor. Means ± standard error of the mean of three independent experiments are shown.

To assess the functional effect of the Ghent +49A > G mutation, the relative binding of recombinant IRP1 to the mutated IRE was evaluated *in vitro* by EMSA experiments. Qualitative results obtained by direct EMSA experiments indicate a 75% decrease (mean of four experiments) for binding of the Ghent +49A > G mutation to IRP1, relative to the WT *FTL* IRE sequence (Fig. 3A). As expected, the positive control +39ΔC mutation showed only minimal binding to IRP1, with a reduction of almost 95%. The full-length gel image is included in Supplementary Figure 3. One-way ANOVA showed that differences in the mean of intensities for the Ghent +49A > G mutation, negative and positive control are statistically significant (Fig. 3B). *Post hoc* analysis indicated that the mean of all three groups are statistically different from each other. The effect of the Ghent +49A > G mutation on IRP1 binding was evaluated quantitatively using a more stringent assay, a competitive EMSA. Using this assay, it was demonstrated that the +49A > G mutant competitor is less efficient in displacing the labeled probe than the WT competitor, as observed by a more gradual disappearance of the shifted band on the gel in the presence of increasing amounts of +49A > G mutant competitor when compared to the WT competitor (Fig. 4A). In particular, the competitor containing the Ghent +49A > G mutation starts to compete effectively at concentrations between 10- and 20-fold excess, which is significantly later than the negative control WT competitor that begins to show significant competition between 2- and 5-fold excess. In contrast, the +39ΔC competitor, used as a positive control, is inefficient in displacing the labeled probe, since the IRE containing this mutation does not show significant competition even at 80-fold excess. Full-length gel images are included in Supplementary Figure 4. A plot of the quantified band intensities in function of the molar excess of the competitor is given by Fig. 4B.

Quantification of the apparent dissociation constant (K_Dapp_) of the WT and +49A > G mutant IRE using a single independent site model^21^ indicates that the WT IRE has an K_Dapp_ of 0.50 nM, while the +49A > G mutant IRE demonstrates a more than 5-fold lower affinity for IRP1, with an K_Dapp_ of 2.70 nM. Since the +39ΔC mutant IRE is ineffective at binding IRP1, no single independent site model could be fitted to the values of this mutant IRE, therefore no K_Dapp_ could be calculated. In summary, *in vitro* EMSA experiments support a decreased binding affinity of the +49A > G mutant IRE for IRP1.

## Discussion

In this study a novel mutation in the IRE of *FTL*, Ghent +49A > G, was found in a homozygous and heterozygous state in different members of a consanguineous family with HHCS. Another mutation at the same nucleotide position (+49A > C) has been reported once in a family with HHCS, however, no clinical data were provided^23^. So far, more than thirty point mutations and six deletions in the *FTL* IRE have been identified in patients with HHCS^24,25^. The Ghent +49A > G mutation adds to the repertoire of HHCS causing mutations.

The zygosity of +49A > G was found to correlate with disease expression, with the most severe phenotype observed in the index case harbouring the mutation in homozygous state. In particular, the serum ferritin level of the proband displays a roughly 5-fold increase in comparison with the heterozygous family members and is almost 10-fold higher than the normal reference values. Moreover, despite the young age of the proband, he shows the most severe clinical manifestations of cataracts among the affected individuals. Despite the fact that genotype-phenotype correlations between serum ferritin levels and HHCS severity are difficult to obtain, mainly due to age and gender variability of serum ferritin, our observation is in line with a more severe clinical phenotype in subjects with a homozygous mutation in comparison with heterozygous patients, as reported for multiple other diseases^26^.

So far, three HHCS families with rare homozygous mutations in the IRE of *FTL* have been reported^25,27,28^. In a first homozygous case serum ferritin levels were more than 2-fold higher than in heterozygous patients. The severity of cataracts however showed no correlation with mutation dose^27^. For the second case, no correlation between the state of zygosity and the severity of the clinical phenotype was found in the two homozygous and four heterozygous patients^25^. In the third case, only the proband was investigated, so no comparison could be made with heterozygous cases^28^. As noted previously, HHCS patients with the same mutation can demonstrate phenotypic diversity due to other genetic or environmental factors, concomitant pathologies or age-related penetrance^6^. Indeed, serum ferritin levels show inter- and intra-individual differences since they are affected by age and sex, while the age of cataract onset and its severity can be influenced by differences in diet, exposure to UV light, or substance abuse^10^. There have been, for instance, reports of young children with elevated serum ferritin levels but without cataracts, having the same mutation as their affected relatives with cataract^12^. In the proband reported here, the early-onset cataract formation could be explained by the unusual high levels of serum ferritin, causing a rapid accumulation of crystalline deposits in the lens. Interestingly, it has been demonstrated that endogenous *FTL* transcription is significantly higher in the lens compared to other eye tissues, which is likely to contribute to the pathogenic levels of FTL deposits found in HHCS lenses^29^. Several authors have also attempted to establish a relationship between the severity of the clinical features and the location of the mutation in the IRE structure^10,25,30^. In general, heterozygous mutations disturbing the hexanucleotide loop or the cytosine bulge give rise to strongly increased serum ferritin levels and early-onset bilateral cataract, while mutations affecting the base pairing of the upper or lower stem generally present a more moderate phenotype. The +49A > G mutation is located at the basis of the upper stem, right above the cytosine bulge. In the heterozygous patients, the mutation gives rise to a moderate increase of serum ferritin and mild cataract, supporting the abovementioned genotype-phenotype correlation.

Sanchez *et al*.^31^ performed a series of competitive EMSA experiments to determine IRP1 binding motifs in the IRE of H-ferritin (*FTH*)^31^. The *FTH* 5′ IRE probe containing the adenine to guanine change at the position corresponding to position +49 of the *FTL* 5′ IRE, showed IRP1 binding characteristics comparable to those of the WT probe. This implies that this change in *FTH* causes no loss of IRP1 binding affinity, as opposed to the +49A > G mutation in *FTL*, shown to effectively reduce IRP1 binding. This difference could be explained by the hypothesis the authors derived from additional experiments. Specifically, it was shown that the presence of three or more G-U or U-G wobble base pairs in the *FTH* 5′ IRE motif severely impairs its ability to compete with the WT probe. The WT *FTL* 5′ IRE has two U-G base pairs, compared to only one in the WT *FTH* 5′ IRE. Since +49A > G induces an additional U-G base pair, the threshold of three U-G base pairs is exceeded in mutant *FLT*, but not in mutant *FTH*. The results of the EMSA experiments in our study therefore illustrate that the hypothesis, allowing a maximum of two G-U or U-G wobble base pairs in order to preserve proper binding characteristics, can be broadened from *FTH* to *FTL*.

Bioinformatics tools show that the substitution of adenine by guanine at position +49 in the upper stem of the IRE is predicted to change the secondary structure of the *FTL* IRE, thereby affecting the apical loop as well as causing a loss of the cytosine bulge, elements that are critical for IRP/IRE recognition^9^. Furthermore, previous studies suggest that proper base pairings at the basis (+34/+49) and the top (+38/+45) of the upper stem of the IRE are crucial for obtaining the correct arrangement of nucleotides composing the upper stem and hexanucleotide apical loop^31^.

The functional effect of the +49A > G mutation in the *FTL* IRE on IRP1 binding affinity was evaluated by means of EMSA experiments. The direct as well as competitive EMSA experiments show that +49A > G reduces, but not completely abolishes, the binding with recombinant IRP1. From the EMSA data, we have calculated K_Dapp_ values for the WT and +49A > G IRE. The K_Dapp_ value of the mutant IRE is more than 5-fold lower than WT IRE, which also corresponds with the outcome of the direct EMSA experiment. The observations from both types of EMSA experiments thus suggest that the *FTL* 5′ IRE with +49 A > G is capable of binding IRP1 *in vitro*, albeit with a significantly reduced binding affinity. It seems, however, that the manifestation of the HHCS phenotype is independent of the strength of the mutation in terms of reduction of binding affinity. In particular, mutations that only mildly impair IRP/IRE binding *in vitro*, can also induce biochemical and clinical symptoms^10,25^, illustrating the high sensitivity of the IRP/IRE complex to minor perturbations in affinity.

In conclusion, we identified a novel non-coding mutation,“Ghent +49A > G”, in the IRE of *FTL* causing variable degrees of HHCS in a consanguineous family. The zygosity of the mutation correlated with the severity of the phenotypes in this family. The mutation was predicted to disrupt the secondary structure of the IRE. Functional characterization of the mutation revealed that +49A > G reduces, but does not completely abolishes, the binding to IRP1.

## Electronic supplementary material

Supplementary data
