# Supplementary material for: Functional characterization of a novel non-coding mutation “Ghent +49A > G” in the iron-responsive element of L-ferritin causing hereditary hyperferritinaemia-cataract syndrome
Source: Sci Rep. 2017 Dec 21;7:18025. doi: 10.1038/s41598-017-18326-6 (PMC5740175; doi:10.1038/s41598-017-18326-6)
Supplement: Supplementary file 1 — Supplementary data [file 41598_2017_18326_MOESM1_ESM.pdf]

Functional characterization of a novel non-coding mutation “Ghent +49A>G” in the iron-responsive element of L-ferritin causing hereditary hyperferritinaemia-cataract syndrome

Stijn Van de Sompele[1], Lucie Pécheux[2], Jorge Couso[3,4], Audrey Meunier[5], Mayka Sanchez[3,4,*], Elfride De Baere[1,*]

Supplementary Data

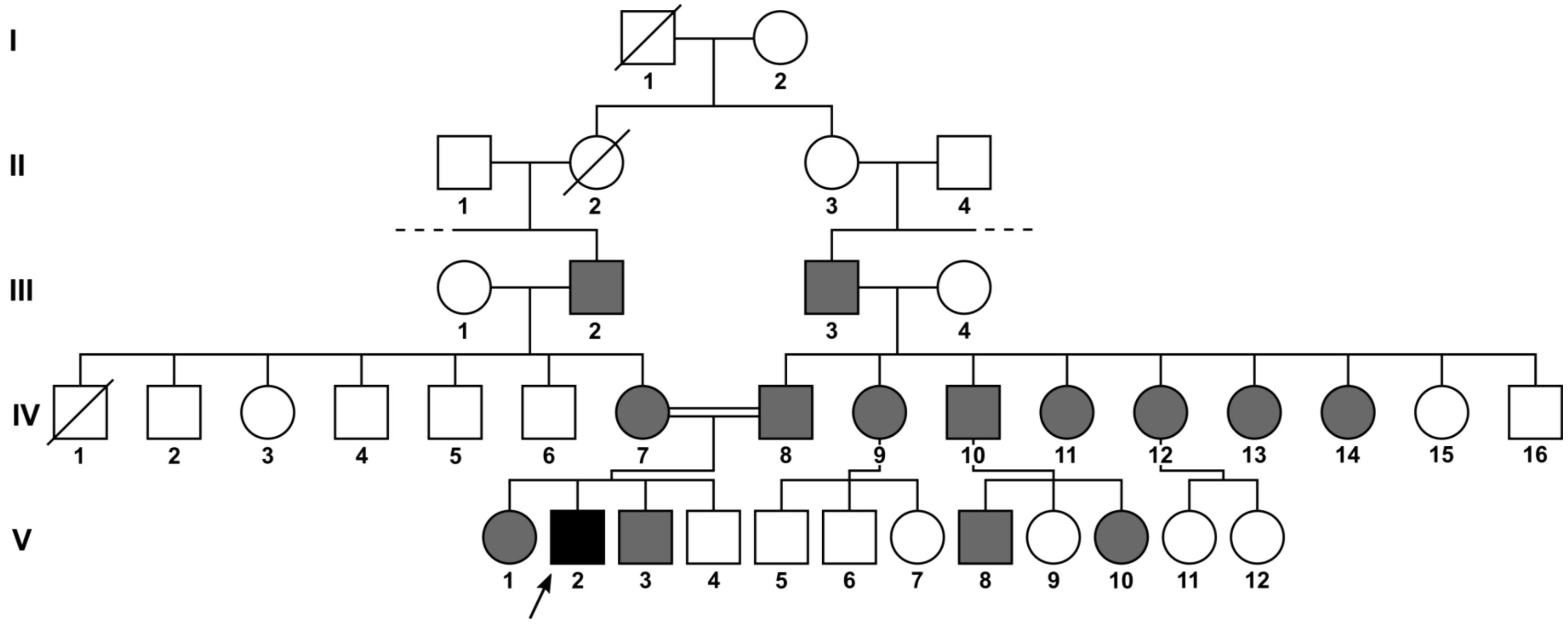

Supplementary Figure 1 - Extended pedigree of the Belgian family of Moroccan descent that was subject of this study, illustrating the consanguineous relationship in the family. The proband (ext_V:2), his parents (ext_IV:7, ext_IV:8) and two of his siblings (ext_V:1, ext_V:3) were clinically and genetically examined. For the other family members, no molecular data was available.

Supplementary Table 1 - Primer sequences used for PCR and Sanger sequencing of the *FTL* exons (NM_000146.3), with an emphasis on the first exon including 5’UTR, indicated in bold, which is relevant in the context of a clinical diagnosis of HHCS. The table below contains primers for copy number analysis of the *FTL* 5’UTR using qPCR.

**PCR**	**Forward (5’-3’)**	**Reverse (5’-3’)**
**Exon 1**	**tgtaaaacgacggccagtTATGTGCTCCGGATTGGT**	**caggaaacagctatgaccATGGAGGCAACAATGGTTAG**
**Exon 2**	tgtaaaacgacggccagtGCGGTCGGGTAAACAG	caggaaacagctatgaccGTTAAGAGGGCTCACAAGAC
**Exon 3**	tgtaaaacgacggccagtTAGTTCTATGTGCCGAGTGT	caggaaacagctatgaccCCTCATCTAGGAAGTGAGTCT
**Exon 4**	tgtaaaacgacggccagtTCATTTCACACCTGTCACAT	caggaaacagctatgaccAACAGATCCCACCTCATCTT

**qPCR**	**Forward (5’-3’)**	**Reverse (5’-3’)**
**Amplicon 1**	actcctatgtgctccggatt	ctagggcggcttcttttatg
**Amplicon 2**	acgtcccctcGCAGTTC	AGATGGCCGAGAAGATGGT
**Amplicon 3**	AACCATGAGCTCCCAGATTC	gggactcacCAGAGAGAGGT

Supplementary Table 2 - Sequences of WT and +49A>G mutant *FTL* 5'UTR IRE RNA used for fold predictions.

**WT *FTL* 5' IRE**
CGGGUCUGUCUCUUGCUUCAACAGUGUUUGGACGGAACAGAUCCG
**+49A>G mutant *FTL* 5' IRE**
CGGGUCUGUCUCUUGCUUCAACAGUGUUUGG**G**CGGAACAGAUCCG

Supplementary Table 3 - Oligonucleotide sequences used as insert for creating the WT, +39ΔC and +49A>G plasmids and primer sequences flanking the insert used for Sanger sequencing.

**WT *FTL* 5' IRE - up**
CGGTCCCGCGGGTCTGTCTCTTGCTTCAACAGTGTTTGGACGGAACAGATCCGGGGACTT
**WT *FTL* 5' IRE - down**
CTAGAAGTCCCCGGATCTGTTCCGTCCAAACACTGTTGAAGCAAGAGACAGACCCGCGGGACCGGTAC

**+39ΔC mutant *FTL* 5' IRE - up**
CGGTCCCGCGGGTCTGTCTCTTGCTTCAA_AGTGTTTGGACGGAACAGATCCGGGGACTT
**+39ΔC mutant *FTL* 5' IRE - down**
CTAGAAGTCCCCGGATCTGTTCCGTCCAAACACT_TTGAAGCAAGAGACAGACCCGCGGGACCGGTAC

**+49A>G mutant *FTL* 5' IRE - up**
CGGTCCCGCGGGTCTGTCTCTTGCTTCAACAGTGTTTGG**G**CGGAACAGATCCGGGGACTT
**+49A>G mutant *FTL* 5' IRE - down**
CTAGAAGTCCCCGGATCTGTTCCG**C**CCAAACACTGTTGAAGCAAGAGACAGACCCGCGGGACCGGTAC

**I-12.CAT forward sequencing primer**
ccagggttttcccagtcac
**I-12.CAT reverse sequencing primer**
ggcatttcagtcagttgctcaatg

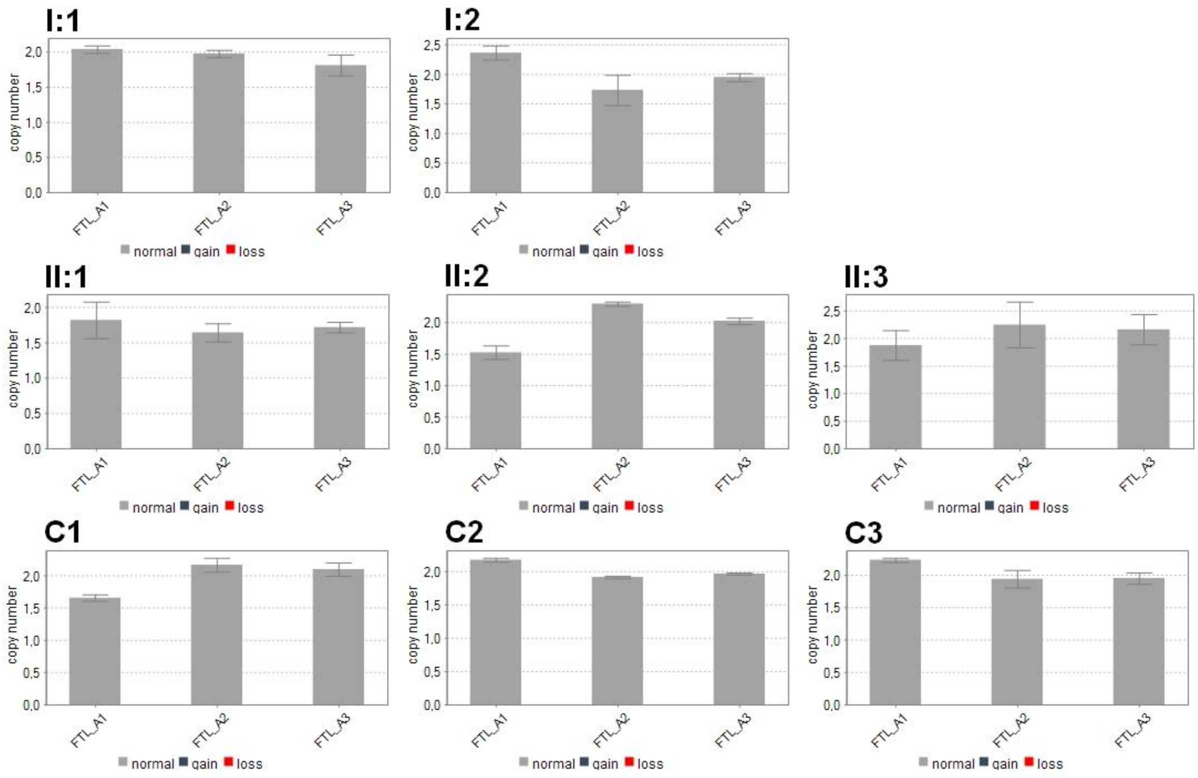

Supplementary Figure 2 - Graphical representation of the qBase+ output. The proband (II:2), the parents (I:1, I:2), two siblings (II:1, II:3) as well as three controls (C1, C2, C3) were each screened for copy number variations in the *FTL* 5'UTR region, by evaluating three qPCR amplicons (A1, A2, A3). All individuals demonstrate two copies for the three amplicons.

Supplementary Table 4 - Overview of regions of homozygosity (> 1 Mb) unique to the index case, obtained by homozygosity mapping using SNP arrays in the index case, the parents and two siblings. The autozygous region containing the *FTL* gene is indicated in bold.

	**Region (GRCh37/hg19)**	**Cytoband**	**Size (bp)**	**Number of genes**
**1**	Chr2:71,162,773-72,309,757	2p13.3	1,146,985	11
**2**	Chr6:54,053,436-55,236,937	6p12.1	1,183,502	5
**3**	Chr7:64,200,641-68,101,280	7q11.21	3,900,640	33
**4**	Chr7:72,214,844-74,077,201	7q11.23	1,862,358	45
**5**	Chr10:21,263,148-23,810,151	10p12.31	2,547,004	22
**6**	**Chr19:49,082,265-51,655,950**	**19q13.33**	**2,573,686**	**247**
**7**	Chr20:3,059,364-4,406,221	20p13	1,346,858	28

Supplementary Table 5 - Overview of the criteria contributing to the variant classification of the *FTL* mutation c.-151A>G (+49A>G) using ACMG 2015 guidelines. **Abbreviations used:** PM: pathogenic moderate; PP: pathogenic supporting; PS: pathogenic strong

**Gene**	***FTL***
**Reference sequence**	NM_000146.3
**g.notation**	g.49468614A>G (GRCh37/hg19)
**c.notation**	c.-151A>G
**p.notation**	p.?
**Population data**	**PM2**: the variant is absent in genomic databases (1000 Genomes Project, ExAC, dbSNP, HGMD)
**Genotype and phenotype of the patient**	**PP4**: the phenotype of the patient and the family history is specific for a monogenic disease caused by mutations in this gene
**Computational predictions**	**PP3**: the majority of the prediction tools predict a deleterious effect
**Genomic location**	**PM1**: the variant is located in a mutational hotspot
**Fuctional data**	**PS3**: functional studies demonstrate a deleterious effect on the mRNA or protein level
**Segregation data**	**PS5**: the variant segregates with disease in several affected family members (# > 4)
**Allelic data**	**PM3**: the variant occurs on both parental alleles (in *trans*)
**Conclusion**	**Pathogenic variant (class 5)**: ≥ 2 strong pathogenic arguments (PS3, PS5)

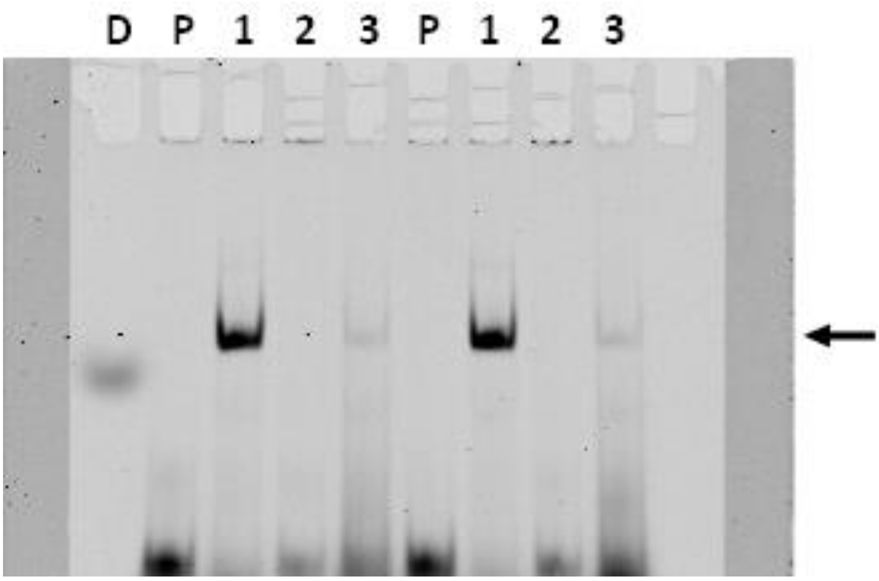

Supplementary Figure 3 - Full-length representative gel image of direct EMSA experiments, without adjustment of brightness and contrast levels. The shifted IRP1/IRE complex is indicated with an arrow. Lane D contains a loading dye. Lane P contains the labeled WT probe in absence of IRP1. Lane 1, 2 and 3 respectively contain labeled WT, +39ΔC mutant and +49A>G mutant probe in the presence of IRP1.

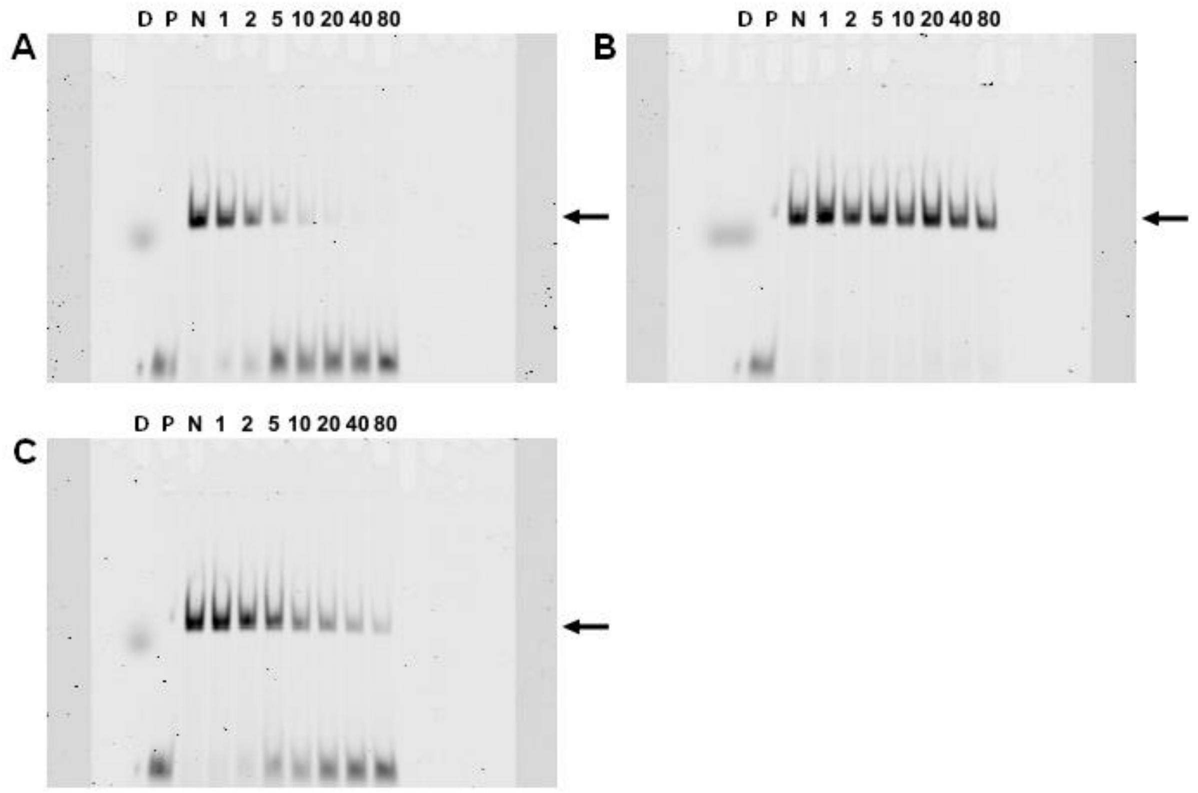

Supplementary Figure 4 - Full-length representative gel images of competitive EMSA experiments, without adjustment of brightness and contrast levels. The shifted IRP1/IRE complex is indicated with an arrow. Lane D contains a loading dye. Lane P contains the labeled WT probe in the absence of IRP1. Lane N contains the labeled WT probe with IRP1 in the absence of unlabeled competitor. The following lanes contain labeled WT probe with IRP1 and increasing amounts of WT (**A**), +39ΔC mutant (**B**) and +49A>G mutant (**C**) competitor.
